# A novel pathway for fungal D-glucuronate catabolism contains an L-idonate forming 2-keto-L-gulonate reductase

**DOI:** 10.1038/srep26329

**Published:** 2016-05-18

**Authors:** Joosu Kuivanen, Maura H. Sugai-Guérios, Mikko Arvas, Peter Richard

**Affiliations:** 1 VTT Technical Research Centre of Finland Ltd, P.O. Box 1000, 02044-VTT, Finland; 2 Departamento de Engenharia Química e Engenharia de Alimentos, Universidade Federal de Santa Catarina, Cx.P. 476 Centro Tecnológico, Florianópolis 88040-900, Santa Catarina, Brazil

## Abstract

For the catabolism of D-glucuronate, different pathways are used by different life forms. The pathways in bacteria and animals are established, however, a fungal pathway has not been described. In this communication, we describe an enzyme that is essential for D-glucuronate catabolism in the filamentous fungus *Aspergillus niger*. The enzyme has an NADH dependent 2-keto-L-gulonate reductase activity forming L-idonate. The deletion of the corresponding gene, the *gluC*, results in a phenotype of no growth on D-glucuronate. The open reading frame of the *A. niger* 2-keto-L-gulonate reductase was expressed as an active protein in the yeast *Saccharomyces cerevisiae*. A histidine tagged protein was purified and it was demonstrated that the enzyme converts 2-keto-L-gulonate to L-idonate and, in the reverse direction, L-idonate to 2-keto-L-gulonate using the NAD(H) as cofactors. Such an L-idonate forming 2-keto-L-gulonate dehydrogenase has not been described previously. In addition, the finding indicates that the catabolic D-glucuronate pathway in *A. niger* differs fundamentally from the other known D-glucuronate pathways.

D-Glucuronic acid is a hexuronic acid derived from D-glucose by the oxidation of C6. It has several essential functions in the cellular metabolism including glucuronidation in xenobiotic metabolism^1^ and being a precursor in L-ascorbic acid biosynthesis^2^. D-Glucuronate occurs also as component of cell wall polysaccharides such as glucuronoxylan^3^ in hemicellulose and ulvan^4^ in algae. D-Glucuronate is catabolised in different life forms through different metabolic pathways. Two bacterial pathways and an animal pathway are known.

In some bacteria, such as *Escherichia coli*, D-glucuronate is in the first step converted by a D-glucuronate isomerase (EC 5.3.1.12) to D-fructuronate which is then reduced by an NADH utilizing D-fructuronate reductase (EC 1.1.1.57) to D-mannonate^5^ (Fig. 1A). In the next step, a D-mannonate dehydratase (EC 4.2.1.8) is removing a water molecule from the D-mannonate producing 2-keto-3-deoxy-D-gluconate^5^. In *E. coli*, the genes for these enzymes are called *uxaC*, *uxuB* and *uxuC*, respectively^6^. The resulting 2-keto-3-deoxy-D-gluconate is phosphorylated to 2-keto-3-deoxy-D-gluconate-6-phosphate which is then split by an aldolase to D-glyceraldehyde-3-phosphate and pyruvate. The genes for these enzymes in *E. coli* are *kdgK* and *kdgA*, respectively^6^. The later part of the pathway is also used in the catabolism of D-galacturonate. The D-galacturonate pathway is very similar; *uxaC*, *kdgK* and *kdgA* are used by both pathways^6^.

A second bacterial pathway for D-glucuronate catabolism (Fig. 1B) exists in which D-glucuronate is oxidised to D-glucaro-1,4-lactone. The lactone is then hydrolysed to D-glucarate which is then converted by a dehydratase to 2-keto-3-deoxy-glucarate^7^,^8^. Alternatively, the lactone is directly converted to the 2-keto-3-deoxy-glucarate by a cycloisomerase^9^. A combined dehydratase and decarboxylase is then producing 2-keto-glutarate-semialdehyde which is then converted in the last step of the pathway by a dehydrogenase to 2-keto-glutarate^8^. Also this pathway can be partly used for D-galacturonate catabolism. The first dehydrogenase, the cycloisomerase and the last two enzymes of the pathway are used for D-glucuronate and D-galacturonate catabolism.

In animals, D-glucuronate is catabolised through a pathway that is sometimes called the glucuronate-xylulose-pentose phosphate pathway or uronate cycle^10^ (Fig. 1C). It was estimated that about 5% of D-glucose is metabolised through this pathway in mammals^11^. The enzymes of this pathway are: D-glucuronate reductase, (EC 1.1.1.19) L-gulonate-3-dehydrogenase (EC 1.1.1.45) 3-keto-L-gulonate decarboxylase (EC 4.1.1.34), L-xylulose reductase (EC 1.1.1.10), xylitol dehydrogenase (EC 1.1.1.9) and xylulokinase (EC 2.7.1.17). The D-glucuronate reductase is an NADPH requiring that was described in human kidney producing L-gulonate^12^. L-Gulonate is then reduced by L-gulonate-3-dehydrogenase to 3-keto-L-gulonate. This enzyme activity has been known for about 50 years^13^ but only recently the corresponding gene was identified^14^. The 3-keto-L-gulonate decarboxylase is then acting on the 3-keto-L-gulonate to produce L-xylulose. A gene coding for a protein with this enzyme activity has not been described. L-Xylulose is then reduced to xylitol by an L-xylulose reductase. The corresponding gene has been identified in animals^15^. The first five reactions of this pathway are shown in the Fig. 1. In the subsequent step D-xylulose is phosphorylated to D-xylulose-5-phosphate which is a metabolite in the pentose phosphate pathway.

In fungal microorganisms, a pathway for D-glucuronate catabolism has not been described, although many fungi are able to catabolise D-glucuronate^16^. However a pathway for D-galacturonate catabolism has been described (Fig. 1D). In this pathway, D-galacturonate is first reduced to L-galactonate by an NADPH requiring reductase^17^. This enzyme is unspecific and can use also D-glucuronate as a substrate generating L-gulonate. After D-galacturonate is reduced to L-galactonate, a dehydratase is active on the L-galactonate to produce 2-keto-3-deoxy-L-galactonate^18^. This is followed by an aldolase to form pyruvate and L-glyceraldehyde^19^. In the last step L-glyceraldehyde is reduced to glycerol^20^.

In the present manuscript we describe an enzyme that is essential for D-glucuronate catabolism in the filamentous fungus *Aspergillus niger*. The enzyme has an NADH dependent, L-idonate forming, 2-keto-L-gulonate reductase activity. The finding indicates that the catabolic D-glucuronate pathway in *A. niger* is different from the bacterial and animal pathway. It is also different from the fungal D-galacturonate pathway, although the first enzyme may be shared between the pathways.

## Results

Since the pathways for D-glucuronate and D-galacturonate catabolism are similar in bacteria we tested whether also in fungi similar pathways are used for these two hexuronic acids. In the first step of the fungal D-galacturonate pathway D-galacturonate is reduced to L-galactonate by an NADPH requiring reductase, which is called Gar1 in *Trichoderma reesei*^17^ and GaaA in *Aspergillus niger*^21^. These enzymes are unspecific and can also reduce D-glucuronate to L-gulonate. The homology in protein sequences between the Gar1 in *T.reesei* and GaaA in *A.niger* is low; however *A. niger* has a gene encoding a close homologue of Gar1 while *T. reesei* has a gene encoding a homologue for GaaA. In order to test whether the *gaaA* or the *gar1* homologue gene is induced on D-glucuronate and to identify the other genes that are involved in the catabolism of D-glucuronate, we made transcription analysis in *A. niger* during growth on D-glucuronate. The complete mRNA was sequenced after 0, 4 and 20 h after shifting fresh mycelia to D-glucuronate as a sole carbon source. In addition, a similar transcription analysis was carried out after 5 hour cultivation on D-galacturonate medium. The results are in the Table 1.

**Figure 2:**
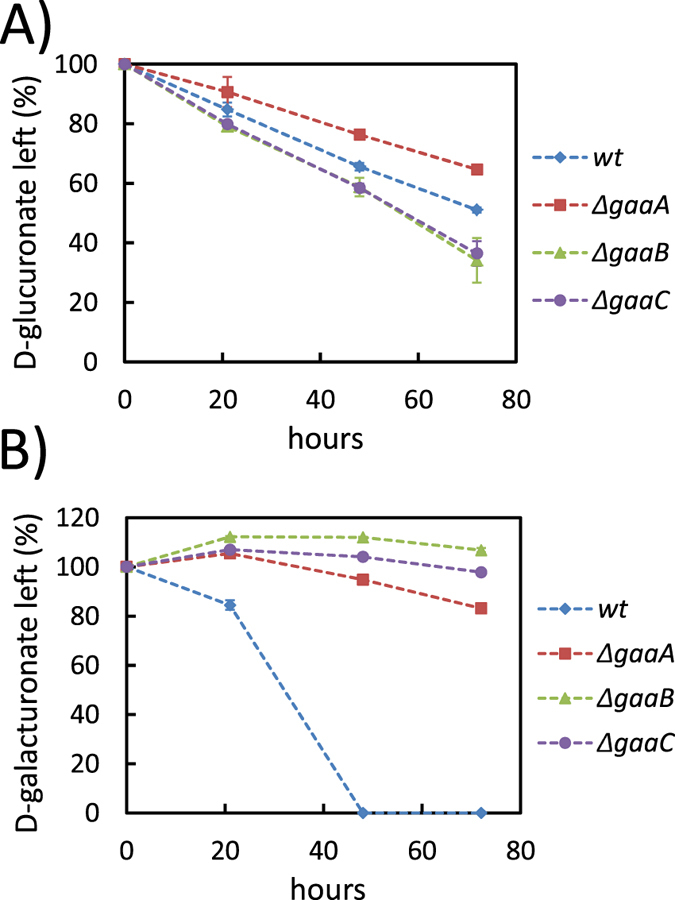
Consumption of (**A**) D-glucuronic acid and (**B**) D-galacturonic acid in submerged cultivations by the *A. niger* strains *wt*, ∆*gaaA*, ∆*gaaB* and ∆*gaaC*. Data represent means ± standard deviation from three biological repeats. If error bars are not visible they are smaller than the symbol.

The *gar1* homologue is not induced whereas the transcription of the *gaaA* is induced on both carbon sources. Since the *gaaA* was upregulated on D-glucuronate we tested a *gaaA* deletion mutant for the utilization of D-glucuronate (Fig. 2A) and D-galacturonate (Fig. 2B) in submerged cultivations. The Δ*gaaA* had a slightly reduced utilization rate of D-glucuronate while the phenotype was more pronounced on D-galacturonate. The observation of a reduced D-glucuronate utilization rate suggests that the *gaaA* may be part of the D-glucuronate pathway. However, the *gaaA* deletion neither blocked the utilization of D-glucuronate nor D-galacturonate completely. Thus, most likely there is an additional reductase(s) functioning in these pathways. We tested also other mutants of the D-galacturonate pathway: The Δ*gaaB* (L-galactonate dehydratase) and Δ*gaaC* (2-keto-3-deoxy-L-galactonate aldolase) strains. In the wild type strain, these genes were induced on D-galacturonate while the induction was lower on D-glucuronate (Table 1). The Δ*gaaB* and Δ*gaaC* strains did not utilize D-galacturonate (Fig. 2B). The observed D-galacturonate concentrations were slightly increasing most likely due to the evaporation during the cultivations. In contrast, the utilization of D-glucuronate was not reduced (Fig. 2A) suggesting that *gaaB* and *gaaC* are not involved in the D-glucuronate pathway. In a previous study all the genes of the *A. niger* ATCC 1015 that had some homology to a sugar acid dehydratase, including *gaaB*, were expressed in yeast and the activities tested with a small library of sugar acids^22^. Since none of these dehydratases exhibited activity with L-gulonate we concluded that there must be some other enzyme activity to convert L-gulonate.

In the RNA sequence analysis a gene with the JGI protein ID 1220513^23^ was upregulated in D-glucuronate but not in D-galacturonate (Table 1). The gene putatively codes for an enzyme of the protein family of D-isomer specific 2-hydroxyacid dehydrogenases. The open reading frame of the gene, as predicted in JGI genome database^23^, was ordered as custom synthesized gene and expressed on a multicopy plasmid in *S. cerevisiae*. A few sugar acids including L-gulonate were tested for the activity with the resulting yeast crude extract. In the extract, the protein did not show L-gulonate dehydrogenase activity; however we found 2-keto-L-gulonate reductase activity as well as L-idonate dehydrogenase activity. The activity was strictly dependent on the cofactors NAD^+^/NADH and no activity was observed with NADP^+^/NADPH. We called the gene encoding this enzyme *gluC*.

**Figure 3:**
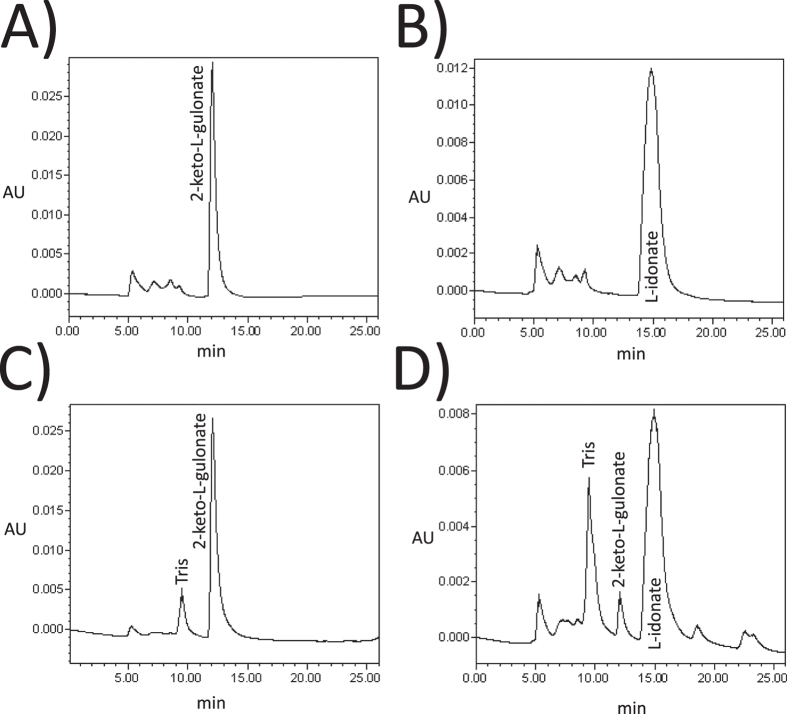
HPLC analysis of (**A**) 10 mM 2-keto-L-gulonate, (**B**) 10 mM L-idonate, (**C**) *in vitro* reaction mixture without GluC and (**D**) *in vitro* reaction mixture with purified GluC.

**Figure 4:**
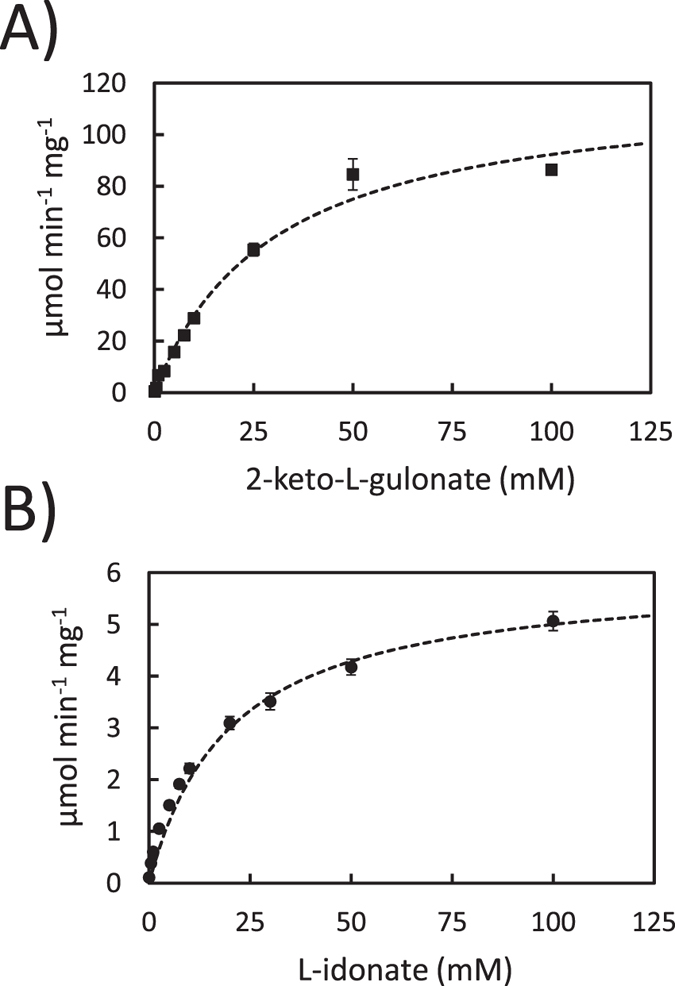
(**A**) 2-keto-L-gulonate reductase and (**B**) L-idonate dehydrogenase activity of the purified GluC with NADH/NAD^+^. Data represent means ± standard deviation from three technical repeats. If error bars are not visible they are smaller than the symbol.

As the next step, the histidine-tagged GluC protein was purified and analysed with more detail. The reaction product of the purified protein was confirmed from *in vitro* reactions containing 2-keto-L-gulonate as substrate using HPLC (Fig. 3) The reaction product was indeed L-idonate and not L-gulonate (L-gulonate has a retention time at 16.2 min). We concluded that the *gluC* codes for a NAD^+^ specific L-idonate forming 2-keto-L-gulonate reductase. Next the kinetic parameters of the purified GluC protein were determined for 2-keto-L-gulonate (Fig. 4A) and L-idonate (Fig. 4B). The V_max_ values 120 and 6 μmol min^−1 ^mg^−1^ and the K_m_ values 30 and 20 mM were determined for 2-keto-L-gulonate and L-idonate, respectively.

**Figure 5:**
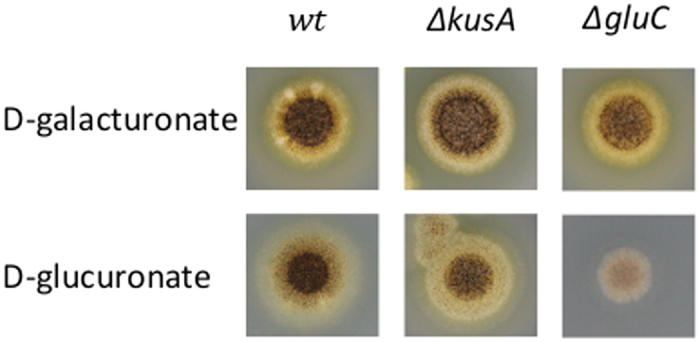
Growth of the *A. niger* strains *wt*, ∆*kusA* and ∆*gluC* on D-galacturonic and D-glucuronic acid on agar plates.

**Figure 6:**
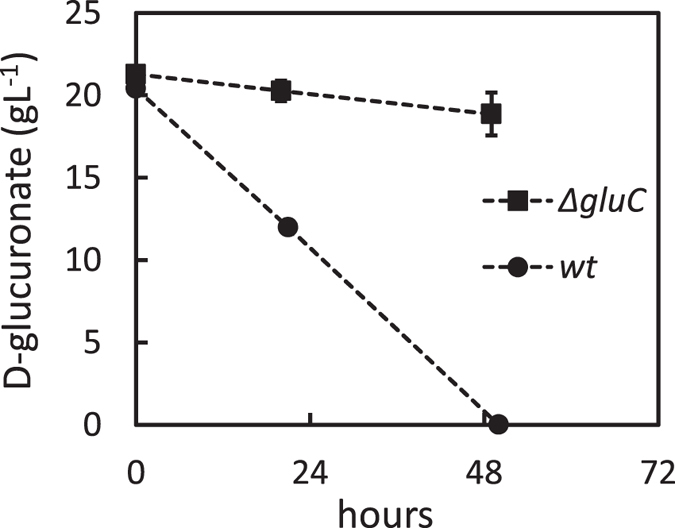
Consumption of D-glucuronic acid in submerged cultivations by *wt* and ∆*gluC*. Data represent means ± standard deviation from three biological repeats. If error bars not visible are smaller than the symbol.

The gene *gluC* was deleted from *A. niger* for the phenotypic characterization of the mutant strain. The resulting ∆*gluC* strain was tested for growth on D-glucuronate containing agar plates (Fig. 5) and for utilization of D-glucuronate in submerged cultivations (Fig. 6). The ∆*gluC* showed only minor growth on D-glucuronate plate probably due to the amino acid supplementation. In the liquid cultivations, utilization of D-glucuronate was almost completely blocked. A slight consumption of D-glucuronate was observed in the submerged cultivation indicating that there may be an additional enzyme for the reaction. However, these results suggest that GluC is the main enzyme for this reaction in the pathway for D-glucuronate catabolism in *A. niger*.

## Discussion

Several fungal species are able to catabolise D-glucuronate; however they are apparently not using any of the bacterial pathways. Genes encoding homologous enzymes for the bacterial pathways are not found in the fungal genomes. Since there is no other information about a fungal pathway for D-glucuronate catabolism, we investigated whether fungi would use the animal pathway. The first enzyme, the D-glucuronate/D-galacturonate reductase, seemed to be part of the fungal pathway, the corresponding gene *gaaA* was upregulated on D-glucuronate and the deletion mutant had a reduced D-glucuronate consumption rate. The L-gulonate that is formed in this reaction is in the next step reduced to 3-keto-L-gulonic acid in the animal pathway. We did not find this enzyme activity in the fungal extract. Another possible path would have been an analogous pathway for the fungal D-galacturonate pathway. In the bacterial pathways, D-galacturonate and D-glucuronate are catabolised using partly the same enzymes. However, we could show that the next two enzymes from the fungal D-galacturonate pathway are not part of the catabolic D-glucuronate pathway in *A. niger*. We also knew from a previous study that *A. niger* does not have a L-gulonate dehydratase^22^. In order to identify genes that are involved in the D-glucuronate catabolism, we made an RNA sequencing. Among the most upregulated genes during growth on D-glucuronate was the *gluC* gene encoding a 2-keto-L-gulonate reductase. This gene was essential for D-glucuronate catabolism in *A. niger* indicating that the catabolic D-glucuronate pathway differs fundamentally from the fungal catabolic D-galacturonate pathway and from other known D-glucuronate pathways. The 2-keto-L-gulonate reductase that forms L-idonate is also an enzyme activity that has not been reported before. A bacterial enzyme had been described previously that converts 2-keto-D-gluconate to D-gluconate. This enzyme is very unspecific and can catalyse also the reaction from 2-keto-L-gulonate to L-idonate using NADPH as a cofactor^24^. The reductase identified in this communication is the first specific L-idonate forming 2-keto-L-gulonate reductase and the first L-idonate forming 2-keto-L-gulonate reductase utilizing NADH. An L-idonate forming 2-keto-L-gulonate reductase had been suggested to be in the plant pathway for L-ascorbic acid conversion to L-tartaric acid^25^,^26^, however the corresponding gene has not been identified.

**Figure 7:**
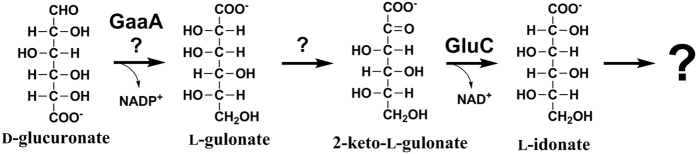
First three suggested reactions in the catabolic D-glucuronate pathway in *A. niger*. The enzymes are: the hexuronate reductase (EC 1.1.1.19) GaaA and the 2-keto-L-gulonate reductase GluC.

For the fungal pathway for D-glucuronate catabolism, we suggest that the first reaction is a reduction producing L-gulonate. However, deletion of *gaaA* did not block the catabolism of D-glucuronate nor D-galacturonate completely. Thus, there must be an additional enzyme(s) having this reductase activity. The *gaaA* is the only gene that is shared by the two pathways. In the D-glucuronate pathway, L-gulonate is most likely then converted to 2-keto-L-gulonate, however there is no information about this step and whether it is a single step. The 2-keto-L-gulonate is then converted to L-idonate by the action of GluC. A metabolic pathway for L-idonate catabolism is described in *E. coli*^27^. In this pathway, L-idonate is oxidized to 5-keto-D-gluconate and further reduced to D-gluconate and phosphorylated to 6-phosphogluconate which can be catabolized through Entner-Doudoroff or pentose phosphate pathway. In fungi, no information is available about the reactions after the L-idonate. Nevertheless, the current study is a strong indication that the catabolic D-glucuronate pathway in *A. niger* starts with the reduction to L-gulonate and rearrangement of its hydroxyl group at C2 via 2-keto-L-gulonate resulting in formation of L-idonate (Fig. 7).

## Methods

### Strains

The *Aspergillus niger* strain ATCC 1015 (CBS 113.46) was used as a wild type. The *A. niger* gene deletion strains ∆*pyrG* (deleted orotidine-5′-phosphate decarboxylase), ∆*gaaA* (deleted D-galacturonate reductase), ∆*gaaB* (deleted L-galactonate dehydratase) and ∆*gaaC* (deleted 2-keto-3-deoxy-L-galactonate aldolase) were described earlier^28^,^29^,^30^. All the plasmids were produced in *Escherichia coli* TOP10 cells. The *Saccharomyces cerevisiae* strains ATCC 90845 and a modified CEN.PK2 (*MATα, leu2-3*/*112, ura3-52, trp1-289, his3*-∆*1, MAL2-8*^*c*^*, SUC2*) were used in the homologous recombination for the plasmid construction and for the production of the purified GluC enzyme, respectively.

### Media and cultural conditions

Luria Broth culture medium supplemented with 100 μg ml^−1^ of ampicillin and cultural conditions of 37 °C and 250 rpm were used for *E. coli* cultures. YPD medium (10 g yeast extract l^−1^, 20 g peptone l^−1^ and 20 g D-glucose l^−1^) was used for yeast pre-cultures. After the transformation of an expression plasmid in yeast, SCD-URA (uracil deficient synthetic complete media supplemented with 20 g D-glucose l^−1^) plates were used for uracil auxotrophic selection. SCD-URA medium was used in protein production. All the yeast cultivations were carried out at 30 °C and the liquid cultivations at 250 rpm. *A. niger* spores were generated on potato-dextrose plates and ~10^8^ spores were inoculated to 50 ml of YP medium (10 g yeast extract l^−1^, 20 g peptone l^−1^) containing 30 g gelatin l^−1^ for pre-cultures. Mycelia were pre-grown in 250-ml Erlenmeyer flasks by incubating overnight at 28 °C, 200 rpm and harvested by vacuum filtration, rinsed with sterile water and weighted. In *A. niger* transformations, SCD-URA plates supplemented with 1.2 M D-sorbitol and 20 g agar l^−1^ (pH 6.5) were used. *A. nidulans* defined minimal medium^31^ was used in the *A. niger* cultivations. The minimal medium used in the phenotypic characterization in liquid cultivations (Fig. 2) contained 20 g D-galacturonate l^−1^ or 20 g D-glucuronate l^−1^ and the pH was adjusted to 5. These cultivations were inoculated with 4 g l^−1^ (wet) of pre-grown mycelia. Similar minimal medium was used in the transcriptional analysis but the pH was adjusted to 6.8 and cultivations were inoculated with 13 g l^−1^ (wet) of pre-grown mycelia. In the phenotypic characterization of *A. niger* ∆*gluC* in liquid cultivations (Fig. 5), similar minimal medium containing D-glucuronate was used but the pH was adjusted to 3. These cultivations were inoculated with 17.5 g l^−1^ (wet) of pre-grown mycelia. Agar plates used for phenotypic characterization of ∆*gluC* (Fig. 5) contained SC-medium (synthetic complete), 15 g agar l^−1^ and 20 g D-glucuronate l^−1^ or 20 g D-galacturonate l^−1^. These plates were inoculated with 1.5^*^10^6^ spores.

### Transcriptional analysis

Samples of 2 ml were collected from the cultures and the mycelium was harvested by vacuum filtration. The filtered mycelium was frozen with liquid nitrogen and stored at −80 °C. Total RNA was extracted using the RNeasy Plant Mini Kit (Qiagen). RNA library preparation and sequencing was carried out by GATC (Constance, Germany) using the InView^TM^ Transcriptome Explore package. In brief, a random primed library was prepared for each of the samples and sequenced with Illumina HiSeq 2500 for 50 bp. Read data was trimmed and quality controlled with FastQC (http://www.bioinformatics.babraham.ac.uk/projects/fastqc/). Reads were aligned with tophat2^32^ to *A. niger* ATCC1015 genome version 4.0^23^ retrieved with GFF annotations from http://genome.jgi.doe.gov/Aspni7/Aspni7.home.html and reads counted with R package “GenomicFeatures”^33^.

### Protein production and purification

The yeast codon optimized *gluC* gene (GenScript, USA) was released with *EcoRI* and *BamHI* (both NEB) and was ligated into a modified pYX212 plasmid^34^ containing *TPI1* promoter and *URA3* selectable marker. A yeast strain was then transformed with the resulting plasmid using the lithium acetate method^35^. The yeast strain expressing *gluC* in the plasmid was cultivated overnight on SCD-URA medium in 1000 ml total volume. Cells were collected by centrifugation and washed. The cell pellet was resuspended to 25 ml of lysis buffer (The QIAexpressionist, Qiagen) containing protease inhibitor (Roche), disrupted five times for 30 seconds using glass beads and BeadBeater (Biospec Products). The extract was centrifuged for 40 min at +4 °C and the supernatant was run through a nickel-nitrilotriacetic acid column (Qiagen). The histidine-tagged GluC protein was purified following the QIAexpressionist protocol (Qiagen). The concentration of purified GluC protein was measured with a protein assay kit (Bio Rad).

### Enzymatic assays

The oxidoreductase activity of purified GluC was assayed using Konelab 20XT Clinical Chemistry Analyzer (Thermo Scientific). The reaction mixture contained 50 mM Tris buffer, 400 μM NAD^+^ or NADH, a substrate in different concentrations and purified GluC in a final concentration of 3.6 mg l^−1^. The pH 8 was used with NAD^+^ and L-idonate (Omicron Biochemicals Inc, USA) and pH 7 with NADH and 2-keto-L-gulonate (Omicron Biochemicals Inc, USA). The reaction was started by addition of the purified GluC and the formation/consumption of NADH was followed at 340 nm. For the HPLC analysis, a similar reaction mixture but 10 mM NAD^+^/NADH and L-idonate/2-keto-L-gulonate were used.

### Gene deletions in *A. niger*

For the deletion of the *kusA* gene (an ortholog of the Ku70 protein in other eukaryotes), a component of non-homologous end joining pathway, a deletion cassette containing homologous 5′ and 3′ flanks (~1.5 kb) for targeted integration and the selectable marker *pyrG* (*A. niger*) was constructed. The 5′ and 3′ flanks were amplified by PCR (KAPA HiFi DNA polymerase, Kapa Biosystems) with the primers P1/P2 and P3/P4, respectively (Table 2). The amplified flanks and *pyrG* were joined using assembly PCR and the resulting cassette was ligated into pCR®-Blunt II-TOPO (Invitrogen). The cassette was linearized with *BcuI* (Fermentas) and transformed to *A. niger* ∆*pyrG* strain using the protoplast transformation method and selected for growth in the absence of uracil. Correct integration of the transformed cassette into the genome was confirmed with colony PCR using Phire direct PCR kit (Thermo Scientific) and the primers P13/P14. The *pyrG* gene that was used as selectable marker was deleted again from the ∆*kusA* strain (*pyrG*∆, *kusA*::*pyrG*) as described by Mojzita *et al.*^28^. The resulting strain ∆*kusA* ∆*pyrG* was used as parental strain for the *gluC* deletion. The *gluC* deletion cassette contained homologous 5′ and 3′ flanks (~1 kb) for targeted integration and the selectable marker *pyrG*. The 5′ and 3′ flanks were amplified by PCR with the primers P5/P6 and P7/P8, respectively, pRS426 plasmid with P9/P10 and *pyrG* with P11/P12. The resulting PCR amplified fragments (5′ flank, 3′flank, *pyrG* and pRS426) contained 40 bp compatible ends for homologous recombination. All the fragments were joined using yeast homologous recombination as described earlier^36^. The resulting cassette was linearized with *NotI* (NEB), transformed to *A. niger* ∆*kusA* ∆*pyrG* strain and mutants with successful integration were selected for growth in the absence of uracil. Resulting transformants were screened for the correct integration of the deletion cassette with colony PCR using Phire direct PCR kit (Thermo Scientific) and the primers P15/P16.

### Chemical analyses

Samples of 2 ml were removed from liquid cultivations at intervals and mycelium was separated from the supernatant by centrifugation. The concentration of D-glucuronate and D-galacturonate (from cultivations) or L-idonate and 2-keto-L-gulonate (from *in vitro* reactions) was determined by HPLC using a Fast Acid Analysis Column (100 mm × 7.8 mm, BioRad Laboratories, Hercules, CA) linked to an Aminex HPX-87H organic acid analysis column (300 mm × 7.8 mm, BioRad Laboratories) with 5.0 mM H_2_SO_4_ as eluent and a flow rate of 0.5 ml min^−1^. The column was maintained at 55 °C. Peaks were detected using a Waters 2487 dual wavelength UV (210 nm) detector.

## Additional Information

**How to cite this article**: Kuivanen, J. *et al.* A novel pathway for fungal D-glucuronate catabolism contains an L-idonate forming 2-keto-L-gulonate reductase. *Sci. Rep.* **6**, 26329; doi: 10.1038/srep26329 (2016).

## Figures and Tables

**Figure 1:**
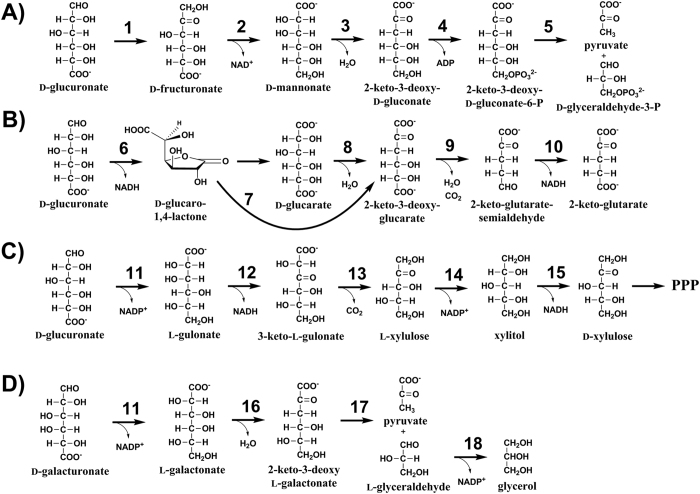
The first reactions of the D-glucuronate catabolism in (**A**) bacterial isomerase pathway, (**B**) bacterial oxidative pathway, (**C**) animals and (**D**) D-galacturonate catabolism in fungi. The enzymes are: (1) D-glucuronate isomerase (EC 5.3.1.12), (2) D-fructuronate reductase (EC 1.1.1.57), (3) D-mannonate dehydratase (EC 4.2.1.8), (4) 2-keto-3-deoxy-D-gluconate kinase (EC 2.7.1.45), (5) aldolase (EC 4.1.2.25), (6) uronate dehydrogenase (EC 1.1.1.203), (7) cycloisomerase (EC 5.5.1.-), (8) dehydratase (EC 4.2.1.40), (9) decarboxylating 2-keto-3-deoxy-glucarate dehydratase (EC 4.2.1.41), (10) 2-keto-glutarate semialdehyde dehydrogenase (EC 1.2.1.26), (11) hexuronate reductase (EC 1.1.1.19), (12) L-gulonate 3-dehydrogenase (EC 1.1.1.45), (13) 3-keto-L-gulonate decarboxylase (EC 4.1.1.34), (14) L-xylulose reductase (EC 1.1.1.10), (15) xylitol dehydrogenase (EC 1.1.1.9), (16) L-galactonate dehydratase (EC 4.2.1.146), (17) 2-keto-3-deoxy-L-galactonate aldolase (EC 4.1.2.54) and (18) L-glyceraldehyde reductase (EC 1.1.1.372).

**Table 1:** RNA sequencing of *A. niger* wild type strain cultivated on D-glucuronate (D-glcUA) and D-galacturonate (D-galUA).

Transcript level (FPKM)							
Gene	JGI protein ID	Genomic location	D-glcUA			D-galUA	Function/InterPro prediction
			0 h	4 h	20 h	5 h	
*gaaA*	1158309	chr_402:1949209–1950762	77	1618	1371	2563	D-galacturonate/D-glucuronate reductase
*gaaB*	1186088	chr_502:1524792–1526872	104	520	851	4714	L-galactonate dehydratase
*gaaC*	1145855	chr_503:270465–271586	98	506	578	827	2-dehydro-3-deoxy-L-galactonate aldolase
*gaaD*	1127092	chr_701:333409–334937	241	670	1009	2887	L-glyceraldehyde reductase
*gar1*	1108731	chr_502:1395768–1397124	89	59	130	134	Homolog of *T. reesei* D-galacturonate reductase
*gatA*	1202517	chr_102:1065966–1067890	6	184	177	391	D-galacturonate transporter
*gluC*	1220513	chr_601:1159357–1160584	4	1285	645	9	IPR006139: D-isomer specific 2-hydroxyacid DH
–	1013899	chr_304:170864–172922	1015	916	929	1213	IPR004000: Actin/actin-like

Transcript levels are presented as fragments per kilobase of exon per million fragments mapped (FPKM).

**Table 2:** Oligonucleotides used in the study.

Name	Sequence
P1	GATAAGCTTGATATCGAATTCCTGCAGCCCGGGGGATCCACTAGTCTTTGCATCACCGCATGCAC
P2	AGCTGGTATAGCCAAACATCGCCAATCACCTCAATCACCCAGTAGAATGTTGTGGAATCGTTTAAAGC
P3	CATGCGGGCTTGGGACGCCATGTCCGTCGCGTGATAACCCCATGGCGGGATTGTTGGATT
P4	CGGTGGCGGCCGCTCTAGAACTAGTATGTTTCGGCGCACTAATAGC
P5	TGATATCGAATTCCTGCAGCGCGGCCGCCAAGGTTCAGGGATCATGGT
P6	CAATCACCTCAATCACCCGGAGAGGATTTGGAAAGTCAAC
P7	CATGTCCGTCGCGTGATAACCGCACATCGTCACCCATTTC
P8	GCTCTAGAACTAGTGGATCCCCCGGGCGGCCGCCTTTCAGAGAGCGACTCGGC
P9	GCTCTCTGAAAGGCGGCCGCCCGGGGGATCCACTAGTTCT
P10	CCCTGAACCTTGGCGGCCGCGCTGCAGGAATTCGATATCAA
P11	GTTGACTTTCCAAATCCTCTCCGGGTGATTGAGGTGATTG
P12	GAAATGGGTGACGATGTGCGTTATCACGCGACGGACATG
P13	ATACTTCCCTCTTT CAATTTCG
P14	AAGACACCACATAACGACATCC
P15	CTACCAATCCTGGAAGGAGA
P16	AGCTGGTATAGCCAAACATC
